# The Role of Supply Chain Management in Healthcare Waste Management: A Scoping Review on Reverse Logistics, Inventory Control, and Environmental Health Impacts

**DOI:** 10.1002/puh2.70386

**Published:** 2026-09-23

**Authors:** Sikhanyiselwe Nkomo, Maasago Mercy Sepadi, Kabelo Kgarosi, Evans Duah, Kuhlula Maluleke

**Affiliations:** ^1^ Centre for Development and Implementation of Point‐of‐Care Diagnostics School of Health Systems and Public Health Faculty of Health Sciences University of Pretoria Pretoria South Africa; ^2^ Department of Environmental Health Faculty of Sciences Pretoria Campus Tshwane University of Technology Pretoria South Africa; ^3^ Library and Information Services Sefako Makgatho Health Sciences University Pretoria South Africa

**Keywords:** healthcare supply chain management, healthcare waste management, inventory control, regulatory compliance, reverse logistics, waste generation

## Abstract

**Background and Aim:**

Efficient healthcare waste management (HCWM), guided by supply chain management (SCM) principles such as reverse logistics (RL), inventory control, and regulation, is essential for reducing environmental and health risks. However, weak supply chain systems, particularly in low‐resource settings, continue to undermine sustainability. This scoping review maps how SCM influences HCWM globally, focusing on RL and inventory control, their environmental health impacts, and opportunities to strengthen sustainable waste management systems.

**Methods:**

The review followed the PRISMA‐ScR guidelines and Arksey and O'Malley framework, structured using the Population–Concept–Context approach. Literature was searched in Scopus, PubMed, Web of Science, and EBSCOhost. The research questions were as follows: (1) How does SCM influence HCWM globally? (2) What roles do RL and inventory control play in shaping environmental health impacts within healthcare systems? Two reviewers independently screened titles, abstracts, and full texts using Covidence, and study quality was assessed using the Mixed‐Methods Appraisal Tool.

**Results:**

A total of 506 records were identified, of which 21 studies met the inclusion criteria. Findings show that healthcare SCM influences HCWM through interconnected policy, operational, technological, economic, and behavioral domains. Effective inventory control and RL reduce waste and improve resource recovery, whereas weak governance, limited infrastructure, workforce constraints, and low public engagement hinder circular economy (CE) implementation and worsen environmental outcomes.

**Conclusion:**

The findings highlight the critical role of SCM in advancing CE practices by promoting waste reduction, traceability, and environmental sustainability. Strengthening regulations, building capacity, and adopting technological innovations are essential to achieving effective and sustainable HCWM.

## Graphical abstract

Efficient healthcare waste management relies on good supply chain systems to reduce health and environmental risks. This review found that weak policies, poor infrastructure, and limited investment impede progress. Strengthening regulations, training staff, using technology, and involving the public are key to safer, more sustainable handling and disposal of healthcare waste.

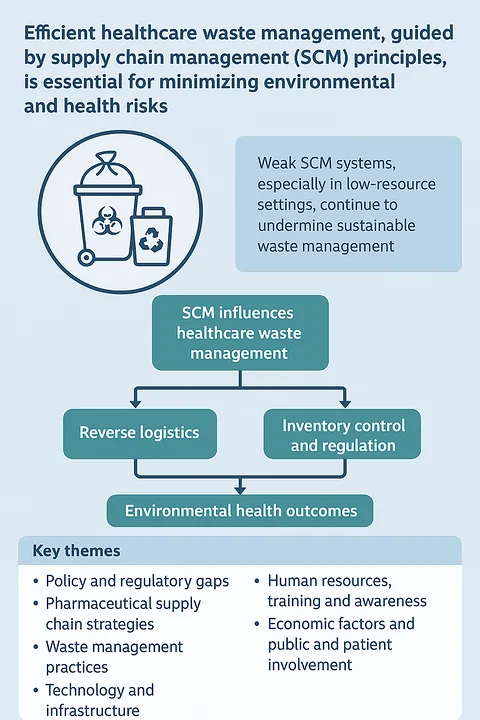

## 1 Introduction

Healthcare supply chain management (SCM) is a critical component of health system performance, involving the planning, procurement, storage, distribution, and disposal of medical supplies and diagnostics [1]. An efficient healthcare SCM ensures timely and continuous access to essential health products while minimizing waste, costs, and environmental hazards [2]. Beyond procurement and distribution, SCM extends to end‐of‐life processes through waste management and reverse logistics (RL), which involves the retrieval, safe handling, and disposal or recycling of expired, damaged, or unused products [3]. Effective healthcare waste management (HCWM) is equally essential for protecting public health, ensuring regulatory compliance, and safeguarding the environment, particularly because improper handling of hazardous medical waste can lead to environmental contamination, antimicrobial resistance, and serious health risks [4]. These risks are particularly pronounced in low‐ and middle‐income countries (LMICs), where weak SCM systems and resource constraints often compromise safe waste management practices. The COVID‐19 pandemic further highlighted this relationship, as the surge in medical waste from personal protective equipment, diagnostics, and pharmaceuticals placed unprecedented pressure on healthcare waste systems globally, demonstrating the need for resilient and sustainable supply chain systems [5, 6].

Globally, healthcare systems operate within regulatory frameworks that mandate the safe handling, segregation, and disposal of healthcare waste, such as the National Environmental Management: Waste Act in South Africa and environmental protection regulations in Ghana [7, 8]. Alongside these regulatory measures, extended producer responsibility (EPR) provides an important approach for linking supply chain activities with waste management beyond the point of use. EPR encourages producers to take greater responsibility for the collection and appropriate management of products at the end of their useful life [9]. In healthcare, this is particularly relevant to pharmaceuticals, medical devices, and packaging, where RL can support the return and proper disposal of unused or expired products [10]. However, implementing EPR globally remains challenging, especially in LMICs due to gaps in regulatory capacity, infrastructure, financing, and enforcement [9, 10, 11, 12].

Despite these frameworks, healthcare facilities continue to face operational inefficiencies, including poor inventory control, stockpiling, over‐ordering, weak coordination for returned or expired products, and inadequate RL systems, all of which contribute to unsafe waste disposal and environmental risks [3, 13]. Although the relationship between SCM and HCWM is increasingly recognized, existing evidence remains fragmented, with limited synthesis of how key supply chain processes such as inventory management, RL, and regulatory compliance collectively influence healthcare waste outcomes. To the best of our knowledge, this is the first global synthesis to examine HCWM through a unified SCM framework that simultaneously integrates RL, inventory management, and regulatory obligations. By bringing these interrelated dimensions together under one coherent analytical umbrella and linking them with environmental health outcomes, this review provides a more holistic understanding of how SCM mechanisms collectively influence HCWM performance and sustainability.

Strengthening SCM has the potential to improve inventory management, enhance regulatory compliance, reduce waste generation, and support sustainable RL systems. These improvements can help healthcare facilities and managers optimize HCWM practices, inform policy and regulatory decisions, and contribute to more sustainable health systems, while aligning with World Health Organization (WHO) strategies and Sustainable Development Goals (SDGs) 3, 12, and 13 on health, responsible consumption, and climate action, respectively [13, 14]. In response to existing evidence gaps, this scoping review aims to map and synthesize global evidence on the influence of healthcare SCM on HCWM, with particular focus on RL, inventory control, regulatory compliance, and environmental health outcomes across healthcare facilities, including hospitals, clinics, laboratories, and pharmaceutical facilities. The review considers all types of healthcare waste generated within these settings while excluding waste streams from non‐healthcare sectors to maintain clear boundaries for the evidence synthesis. Non‐healthcare waste streams, such as general community or public waste, were excluded because their sources, composition, management, and regulatory requirements differ from those of healthcare waste. This ensured that the review remained focused on healthcare supply chain practices and their role in HCWM, consistent with the population, concept, and context (PCC) framework. The review seeks to identify how SCM influences HCWM globally, with particular attention to the roles of RL and inventory control in shaping environmental health impacts within healthcare systems. It also aims to identify existing practices, implementation challenges, policy gaps, and opportunities for strengthening sustainable HCWM systems. It is guided by two research questions: (1) How does healthcare SCM influence HCWM globally? and (2) What roles do RL and inventory control play in shaping environmental health outcomes within healthcare systems?

## 2 Methodology

### 2.1 Rationale

A scoping review design was adopted to map the evidence on how SCM influences HCWM globally, with particular focus on the roles of RL and inventory control in shaping environmental health impacts. A scoping review design was selected because the available evidence is expected to be broad and heterogeneous in terms of study designs, healthcare settings, waste streams, SCM practices, and reported outcomes. Rather than estimating pooled effects or determining the effectiveness of a specific intervention, as would typically be the focus of a systematic review, this review seeks to characterize the breadth, nature, and distribution of existing evidence and identify how different SCM components relate to HCWM. This approach is, therefore, appropriate for concept mapping, identifying implementation challenges and evidence gaps, and highlighting emerging practices and opportunities for strengthening sustainable HCWM systems. The review was guided by the Arksey and O'Malley framework and enhanced by the PRISMA‐ScR reporting guidelines to ensure a transparent, systematic, and reproducible process. These methodological frameworks supported a structured approach to study identification, selection, and evidence charting while minimizing selection and reporting bias.

### 2.2 Review Framework

This scoping review was conducted in accordance with the PRISMA‐ScR guidelines [15]. The methodological framework outlined by Arksey and O'Malley [16] guided the review, ensuring a systematic approach to identifying, selecting, and synthesizing relevant literature. This framework followed a five‐step process for conducting a scoping review: identifying the research question, identifying relevant studies, selecting eligible articles, charting the data and collating, and summarizing and reporting the results.

### 2.3 Identification of the Research Question

The research question was developed iteratively following preliminary literature scoping and was guided by the aim of mapping the influence of SCM on HCWM globally. Due to the broad and complex nature of the topic, the question was refined into two focused sub‐questions to enable structured synthesis of evidence.

- How does SCM influence HCWM globally?
- What roles do RL and inventory control play in shaping environmental health impacts within healthcare systems?

To ensure rigor, transparency, and reproducibility, the research question and eligibility criteria for the data search were defined and structured using the PCC framework recommended by the Joanna Briggs Institute [17]. This is outlined in Table 1.

**TABLE 1:** Population, concept, and context (PCC) framework.

Population	Healthcare facilities (hospitals, clinics, laboratories, and pharmaceutical facilities)
Concept	The role of supply chain management in healthcare waste management, including reverse logistics, inventory control, regulatory compliance, and environmental health impacts
Context	Globally

**FIGURE 1:**
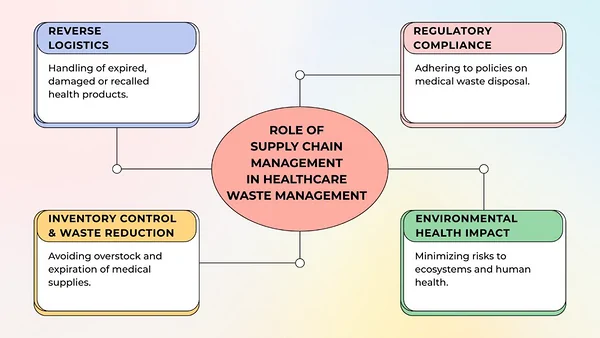
A conceptual framework for the role of supply chain management in healthcare waste management.

Moreover, the concept in the PCC framework was guided by the conceptual framework illustrating the role of SCM in HCWM (Figure 1). This framework highlights four interconnected SCM functions: RL, regulatory compliance, inventory control and waste reduction, and environmental health impact. These functions shape the safe and sustainable handling of medical products and waste. By aligning these elements with the PCC approach, the study ensured a focused and systematic identification of concepts relevant to HCWM.

### 2.4 Identification of Relevant Studies

A comprehensive literature search was conducted without applying any language restrictions, using the following electronic databases: Scopus, PubMed, Web of Science, and EBSCOhost (including Academic Search Complete, Business Source Complete, EconLit, and MEDLINE). An experienced information specialist and librarian collaborated in the development of the search strategy, utilizing relevant keywords and Medical Subject Headings (MeSH) terms, which were appropriately tailored to each database. Additional literature was retrieved from the WHO Global Health Library and national health department websites. These sources were searched to capture policy documents, technical reports, guidelines, and strategic frameworks relevant to SCM and HCWM that may not be available. The search focused on documents published by the recognized health authorities and international organizations, using key terms related to HCWM, SCM, RL, inventory control, regulatory compliance, and sustainability.

#### 2.4.1 Search Strategy

The search strategy combined three major concept groups using Boolean operators:

1. Supply Chain Management Terms: “supply chain management” OR SCM OR “healthcare logistics” OR “medical supply chain” OR “pharmaceutical logistics” OR “inventory control” OR “inventory management”
2. Healthcare Waste Terms: “waste management” OR “medical waste” OR “biohazard waste” OR “hazardous waste sites” OR “infectious waste” OR “medical waste disposal” OR “expired drugs” OR “pharmaceutical waste” OR “environmental health” OR “waste segregation” OR “sharps disposal” OR “solid waste management”
3. Regulatory and Environmental Health Terms: “regulatory compliance” OR “health regulations” OR “environmental health impact” OR “environmental contamination” OR “public health risk” OR “infection control” OR “antimicrobial resistance” OR “pollution”

To identify additional relevant studies, reference lists of all included articles were manually screened. All search results were documented in detail, including keywords, Boolean terms, MeSH terms, date of search, database name, and the number of articles retrieved. The full search strategy and results are presented in Supporting Information (Appendix 1).

## 3 Selection of Eligible Articles

### 3.1 Eligibility Criteria

An extensive title and abstract screening were conducted on the basis of predefined eligibility criteria to determine which articles were eligible for inclusion in this review. The inclusion and exclusion criteria outlined below were applied to ensure that all relevant studies were appropriately considered.

#### 3.1.1 Inclusion Criteria

This review included published records that met the following criteria:

- Focused on HCWM within the SCM framework, including RL, inventory control, regulatory compliance, and environmental health impacts.
- Conducted in healthcare facilities such as hospitals, clinics, diagnostic laboratories, and pharmaceutical facilities worldwide.
- Discussed policies, guidelines, or interventions related to healthcare waste disposal and SCM strategies.
- Published up to the year 2025 to capture recent developments in SCM and HCWM.
- Consisted of peer‐reviewed journal articles, grey reports, or official guidelines from reputable organizations such as the WHO, the United Nations (UN), or national health agencies.

#### 3.1.2 Exclusion Criteria

Studies were excluded from this review if they met any of the following conditions:

- Did not explicitly focus on the intersection of SCM and HCWM.
- Were unrelated to healthcare (e.g., general supply chain logistics outside the medical sector).
- Focused solely on general environmental pollution without a clear connection to HCWM.
- Were opinion pieces, editorials, or non‐peer‐reviewed literature lacking empirical or policy‐based findings.

### 3.2 Data Screening

**FIGURE 2:**
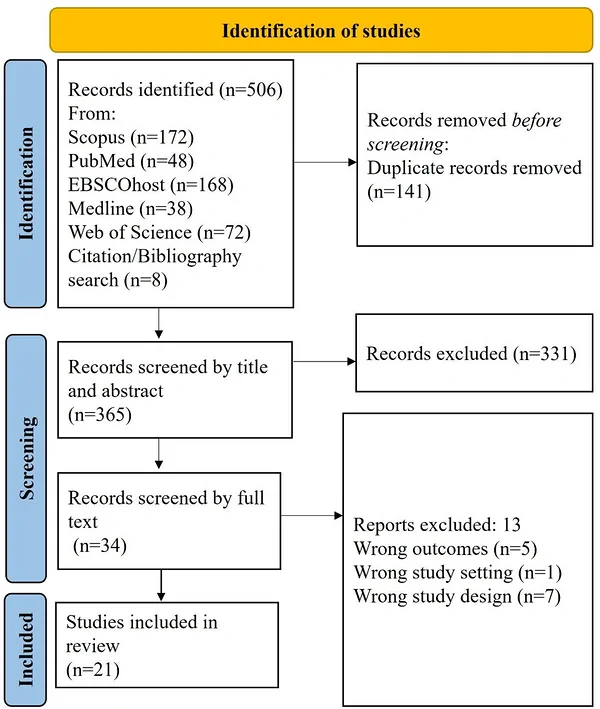
PRISMA‐ScR flow chart showing literature search and selection of records.

Relevant articles were imported into EndNote 20 reference management software, where duplicates were removed. The deduplicated articles were then transferred to Covidence [18] for screening. Two independent reviewers screened the titles and abstracts of the eligible articles based on the predefined inclusion and exclusion criteria. Discrepancies during this stage of screening were discussed between the two reviewers until consensus was reached. Subsequently, the same two reviewers assessed the full texts of the included studies. Any disagreements at this stage were resolved by a third reviewer. An information specialist or librarian assisted in retrieving articles that were not readily accessible during the screening process, for example, articles placed behind paywalls. Moreover, the authors of relevant studies were contacted to provide any missing information identified during the search. The database search process, including search terms, the number of articles retrieved, and those selected for inclusion, was documented. Additionally, the PRISMA‐ScR flow diagram was employed to systematically record the screening process, including reasons for article exclusion (Figure 2).

Inter‐reviewer agreement following full‐text screening was assessed using Cohen's kappa statistic. The analysis yielded a kappa coefficient of *κ* = 0.62 (*n* = 21, SE = 0.20, *p* = 0.0011), indicating substantial agreement between reviewers (Supporting Information, Appendix 2). Interpretation of kappa values followed established thresholds, where values below 0.10 indicate no agreement, 0.10–0.20 slight agreement, 0.21–0.40 fair agreement, 0.41–0.60 moderate agreement, 0.61–0.80 substantial agreement, and 0.81–1.00 almost perfect agreement [19].

### 3.3 Charting the Data

An electronic data charting form was created in the Covidence screening platform using the components of the data extraction tool, which included Article ID, References, aim of study, country, study design, healthcare setting, SCM strategies, waste management practices, and key findings, to extract pertinent information from each of the selected articles.

### 3.4 Quality Appraisal

The methodological quality of the included studies was evaluated using the Mixed‐Methods Appraisal Tool (MMAT) Version 2018 [20], which allows appraisal across qualitative, quantitative, and mixed‐methods study designs. Each study was evaluated against design‐specific criteria, and appraisal outcomes were used to describe the overall methodological quality and rigor of the included evidence. The tool was applied to categorize studies as low quality (score ≤50%), average quality (score 51%–75%), or high quality (score 76%–100%), based on criteria such as the study's relevance, design, methodological adequacy, data collection and analysis, and key findings [20]. All included records achieved quality assessment scores 80%–100%, indicating overall high methodological quality (Supporting Information, Appendix 3).

### 3.5 Collating, Summarizing, and Reporting the Results

The findings were grouped thematically to address the research questions, and a narrative synthesis approach was adopted to summarize emerging evidence on the role of SCM in HCWM. Given the heterogeneity across included studies in terms of study designs, settings, and reported outcomes, findings were synthesized by identifying recurring patterns, thematic similarities, contextual differences, and variations across key domains influencing healthcare SCM and HCWM practices. The synthesis was structured to highlight major trends, implementation challenges, and evidence across core SCM components, including RL, inventory management, regulatory systems, and environmental impact. Where appropriate, tables and graphs were used to present the findings visually and enhance clarity of the synthesized evidence.

### 3.6 Strategies for Minimizing Bias

First, the use of predefined eligibility criteria and standardized procedures limited selection bias, as reviewers did not make ad hoc decisions regarding study inclusion but instead applied the same rules consistently, thereby reducing subjective judgment. Second, applying neutral, non‐value‐laden terminology aligned with PRISMA‐ScR guidelines reduced interpretive and language bias, ensuring that findings were not overstated, understated, or framed in a subjective or judgmental manner. Third, Cohen's kappa (*n* = 21, *κ* = 0.62) was used to address reviewer subjectivity bias. The substantial level of agreement between two independent reviewers indicated that study inclusion decisions were not driven by individual interpretation, demonstrating the reproducibility of decisions as a key safeguard against bias in screening. Finally, reporting the kappa statistic alongside established interpretation thresholds provided transparency and methodological accountability, allowing readers to assess the consistency of the review process.

## 4 Results

### 4.1 Selection of Sources of Evidence

A total of 506 articles were retrieved, comprising 498 from the electronic database search and an additional 8 from the bibliography search, as shown in Figure 2. A total of 365 articles were found eligible for inclusion for abstract and title screening, 141 duplicates were removed. After title and abstract screening, 331 articles were excluded, and 34 met the inclusion criteria for full text screening. Of these, 13 articles were excluded due to the following reasons: 5 records had wrong outcomes, 7 records had wrong study design (e.g., review studies, conference materials, and commentaries), and 1 record had a wrong study setting. Ultimately, 21 records were included and extracted for the review.

### 4.2 Characteristics of Eligible Articles

**FIGURE 3:**
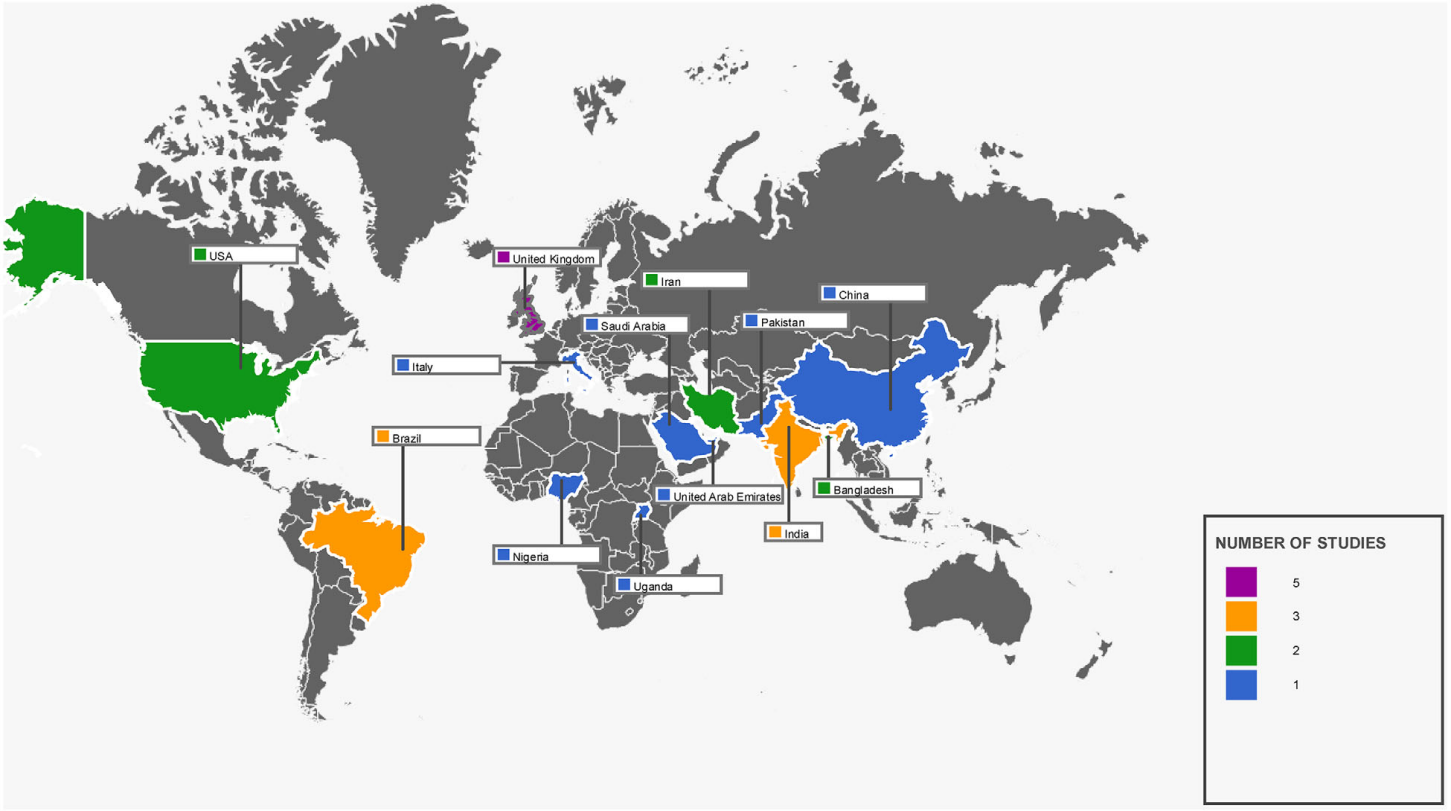
Geographical map of studies.

The characteristics of the eligible articles are summarized in Table 2. The eligible studies were published from 2001 to 2025. The 21 eligible records included 6 qualitative [21, 22, 23, 24, 25, 26], 10 quantitative [27, 28, 29, 30, 31, 32, 33, 34, 35, 36], and 5 mixed‐method [37, 38, 39, 40, 41] studies. The eligible records presented evidence of research conducted in the following countries: Brazil [22, 27, 28], the United Kingdom [32, 37, 38, 39, 41], Kuwait [37], China [21], India [23, 31], Iran [29, 30], Nigeria [35], Bangladesh [33, 40], Italy [25], Saudi Arabia [36], the United States [24, 26], and Pakistan [34] (Figure 3). Most studies were conducted in hospital settings (*n* = 12), followed by those in pharmaceutical settings (*n* = 7), whereas a few were carried out in both environments (*n* = 2). The most commonly discussed waste management strategies were RL and regulatory compliance. Several studies addressed advanced technologies such as radio frequency identification (RFID) and artificial intelligence (AI)‐enabled inventory systems.

**TABLE 2:** Characteristics and findings of the studies included in the scoping review.

Article ID	References	Aim of study	Country	Study design	Healthcare setting	SCM strategies	Waste management practices	Key findings
01	[37]	To identify the enablers and barriers associated with the implementation of circular economy (CE) practices in medicines waste management by hospital pharmacies and to establish whether the implementation of CE principles in medicines waste management may reduce the medicines waste of hospital pharmacies	The United Kingdom and Kuwait	Mixed method	Hospital pharmaceutical	Regulatory compliance, inventory control	Recycling and repackaging	Regulatory frameworks impede the reuse of medicines There is limited public and institutional confidence in reused medications Healthcare professionals exhibit environmental concerns but lack training in circular economy Circular economic principles are not incorporated into professional education or hospital standard operating procedures Opportunities arise through national sustainability objectives and technological integration, particularly in the United Kingdom
02	[27]	To evaluate the effect that critical factors play in the adoption of end‐of‐life management practices for medication and its influence on logistics performance	Brazil	Quantitative	Pharmaceutical	Reverse logistics	Incineration and landfill	Logistics performance plays a crucial role in the management of end‐of‐life (EOL) medicines Key factors influencing EOL of medicines include infrastructure, regulatory compliance, information and communication technology (ICT) integration, training, and collaboration Fragmented data systems and ineffective return mechanisms impede CE strategies for waste alignment Highlights the importance of sustainable logistics and stakeholder involvement in enhancing pharmaceutical EOL processes
03	[28]	To identify the critical factors to the successful implementation of reverse logistics (RL) in the Brazilian pharmaceutical care process (PCP) and determine the cause‐and‐effect relationships among them	Brazil	Quantitative	Pharmaceutical	Reverse logistics	Solid waste management	Identified interdependent and causal factors essential to reverse logistics in pharmaceutical care include: Government policy and regulations, information management systems, financial resources and investment, training, and awareness for staff Integration of decision‐support tools such as Grey‐DEMATEL aids in the prioritization of systemic interventions
04	[22]	To investigate the most relevant critical factors for implementing medicine waste management in pharmaceutical care process	Brazil	Qualitative	Pharmaceutical	Reverse logistics	Municipal‐level disposal	Existing systems do not differentiate between end‐of‐use and end‐of‐life medications, resulting in the wastage of recoverable pharmaceuticals Insufficient public awareness and inadequate return channels contribute to the accumulation of medicines in households and improper disposal practices Pharmacists do not possess the autonomy or regulatory backing necessary to execute reuse or redistribution strategies The integration of circular economy principles into care processes has the potential to optimize resource utilization and mitigate the burden of pharmaceutical waste
05	[23]	To establish an effectiveness index for assessing the performance of biomedical waste management for the Indian states	India	Qualitative	Hospital	Environmental health impact	Waste disposal, recycling, and incineration	A composite effectiveness index was developed to rank states on the basis of performance indicators States such as Kerala and Himachal Pradesh achieved high scores due to superior regulatory compliance and infrastructure States with low scores exhibited deficiencies in treatment facilities and poor segregation practices Key factors for improvement identified include policy enforcement, staff training, and monitoring
06	[38]	To assess how reliably the equipment recommended for a peripheral intravenous care bundle was available for use and to explore factors which contributed to its non‐availability	The United Kingdom	Mixed method	Hospital	Inventory control	Waste disposal	There is inconsistent availability of essential IV insertion equipment across hospital sites Staff frequently improvised due to absent components (e.g., tourniquets and antiseptics) Staff did not have access to sharp bins; hence, they resorted to sending sharps to other locations Patient safety risks increased due to improvisations, delays, multiple attempts, and contamination Significant unintended consequences included high risk of infection, staff stress, and time loss Standardization of pre‐assembled IV insertion packs and enhancements in inventory and waste disposal management are recommended
07	[29]	To analyze the distributors’ corporate social responsibility decisions on collecting unwanted medications under the decentralized and centralized models	Iran	Quantitative	Pharmaceutical	Reverse supply chain, reverse logistics, regulatory compliance	Antibiotic waste disposal	Government involvement via fiscal incentives and regulations is essential for enhancing waste recovery rates and social welfare Decentralized supply chains exhibit reduced efficiency in the absence of coordination Contracts and incentive schemes facilitate the alignment of supply chain actors’ goals with environmental and social objectives Reverse logistics for pharmaceutical waste must incorporate social considerations, such as public health and environmental protection, rather than depend exclusively on economic factors
08	[24]	To highlight Solutions to HealthCare Waste: Life‐Cycle Thinking and “Green” Purchasing	The United States	Qualitative	Hospital	Environmental health impact	Waste disposal, recycling Incineration	Medical waste management is frequently conducted in a reactive manner, with insufficient consideration given to upstream purchasing decisions Life‐cycle thinking identifies environmental impacts at each stage of a product's life from manufacture to disposal Green purchasing significantly reduces hazardous and general waste, energy consumption, and long‐term costs Success is contingent upon institutional commitment, staff training, and the incorporation of environmental criteria into procurement systems
9	[21]	To propose a management model using the reverse logistics based on radio frequency identification (RFID) technology	China	Qualitative	Hospital, pharmaceutical	Reverse logistics	Waste disposal, recycling	RFID systems enhance transparency, efficiency, and safety in medical waste management They facilitate real‐time monitoring and status updates from the point of waste generation to final disposal Additionally, they assist in identifying delays, misrouting, or leakage risks Implementation challenges encompass cost, technical infrastructure, and staff training
10	[30]	To minimize total costs and emission of contamination, simultaneously	Iran	Quantitative	Hospital	Environmental health impact	Waste disposal Incineration	The proposed model addresses two opposing objectives: the minimization of logistics costs and the reduction of contamination risk Stochastic modeling enhances real‐world applicability by incorporating uncertain emission levels Simulation results indicate that the strategic placement of facilities and the implementation of flexible transport routes substantially decrease exposure risk Decision‐makers can balance cost and risk through Pareto‐optimal solutions produced by the algorithm
11	[25]	To review the nature of the raw materials involved in the production, manufacturing techniques and supply chain of medical gowns	Italy	Qualitative	Hospital	Environmental health impact	Waste disposal Recycling	COVID‐19 revealed vulnerabilities in global PPE supply chains, such as excessive dependence on remote suppliers and insufficient surge capacity The post‐COVID recovery should prioritize the equilibrium of safety, sustainability, and resilience Reusable medical gowns have the potential to substantially decrease environmental impact, provided there is adequate sterilization infrastructure and effective procurement policies in place Supports the establishment of integrated procurement standards that encompass environmental and social indicators, in addition to cost and availability
12	[31]	To identify the challenges in biomedical waste (BMW) management in the institute and develop a strategy to improve the knowledge and practices of healthcare professionals (HCPs) in BMW management	India	Quantitative	Hospital	Environmental health impact	Waste disposal	Although awareness of biomedical waste protocols is relatively high, the implementation of these practices remains inconsistent, particularly among support staff Frequent challenges include insufficient training, inadequate supervision, and limited resources Capacity building programs, enforcement of regulations, and access to waste management supplies are essential for enhancing safety and compliance
13	[32]	To standardize the evaluation criteria for the stakeholders to ensure sustainable environmental development by safe disposal of infectious healthcare waste (HCW)	The United Kingdom	Quantitative	Hospital	Regulatory compliance, environmental health impact, reverse logistics	Waste disposal	Critical factors influencing sustainable healthcare waste management consist of government regulations, staff training, funding, technology, segregation, promoted collaboration among multiple stakeholders, improved infrastructure, and the integration of healthcare waste management into national sustainability objectives
14	[26]	To prioritize the identified main group of challenges and their sub‐challenges using the Decision‐Making Trial and Evaluation Laboratory Method (DEMATEL)	The United States	Qualitative	Hospital	Environmental health impact, remanufacturing	Incineration, landfilling	Circular economy provides a practical framework for mitigating the environmental impact of healthcare waste Challenges encompass regulatory limitations, financial constraints, and cultural opposition to reuse Success is contingent upon policy reforms, educational and training initiatives, infrastructure investment, and interdisciplinary collaboration
15	[39]	To develop a novel emission framework to assess the realistic, long‐term emissions of a circular medical device economy	The United Kingdom	Mixed method	Hospital	Environmental health impact, Remanufacturing	Incineration	Remanufactured medical devices generate considerably reduced greenhouse gas emissions over time The long‐term environmental impact is less than that of repeatedly manufacturing new devices The most significant phases in remanufacturing are energy consumption, packaging, and sterilization Policy support and regulatory clarity are crucial for the expansion of remanufacturing in healthcare facilities
16	[33]	To focus on examining the appropriate amount of waste produced due to healthcare services and the current situation of the management system using both quantitative and statistical method	Bangladesh	Quantitative	Hospital	Environmental health impact	Landfill, municipal disposal	Significant deficiencies in infrastructure, regulation, and awareness Predominance of institutions lacking compliance with national biomedical waste regulations Health and environmental hazards resulting from inadequate management and informal disposal methods Necessity for training, enhanced enforcement, investment in treatment technologies, and public‐private cooperation frameworks
17	[34]	To determine waste management practices at selected hospitals in a major city in Pakistan, Gujranwala	Pakistan	Quantitative	Hospital	Environmental health impact	Incineration, landfills, municipal‐level disposal	The majority of hospitals did not achieve full compliance with international requirements for the management of hospital waste Waste was frequently intermixed (hazardous with general) and inappropriately disposed of in municipal landfills Recognized health hazards to employees and adjacent communities resulting from improper disposal methods Recommendations encompassed personnel training, policy implementation, and the establishment of specialized waste treatment facilities
18	[40]	To provide a brief overview of current medical waste generation, compositional analysis of medical waste both worldwide and in Bangladesh	Bangladesh	Mixed methods	Hospital	Environmental health impact	Incineration, microwave sterilization, deep burial	Healthcare waste generation significantly increased during the COVID‐19 pandemic, particularly in urban areas The prevalent mismanagement and open dumping of waste present substantial risks to public health and ecosystems The absence of segregation, protective equipment, training, and enforcement of policies continues to be significant concerns A circular economy approach, which encompasses waste minimization, material recovery, and energy capture, is crucial for achieving long‐term sustainability
19	[35]	To improve the pharmaceutical waste disposal system by assessing the status of pharmaceutical waste disposal among the public in Lagos State	Nigeria	Quantitative	Pharmaceutical	Environmental health impact	Waste disposal	A total of 59.4% of respondents lacked awareness regarding safe pharmaceutical waste disposal practices Many individuals believed that expired or unused medications could be disposed of by flushing them down toilets, throwing them in bins, or giving them to others Most individuals discarded drugs via household trash, without utilizing return or take‐back programs, which are limited in Lagos Sources of medication waste primarily include leftover prescriptions, noncompliance, and the use of over‐the‐counter drugs Respondents demonstrated a significant lack of awareness regarding the environmental and health risks associated with improper disposal
20	[36]	To assess patients’ knowledge and attitude regarding the disposal of medications	Saudi Arabia	Quantitative	Pharmaceutical	Environmental health impact, reverse logistics	Waste disposal	Limited knowledge regarding safe medicine disposal methods A significant proportion of individuals discard medications through household waste or sinks Limited awareness exists regarding take‐back programs or pharmacy return alternatives A considerable number of people keep expired or unused medications at home, heightening the risks of misuse and environmental contamination The propensity for safe disposal is positively correlated with educational attainment and explicit guidance from healthcare professionals There is a perspective that the reuse or donation of medications is permissible, particularly in economically disadvantaged communities Identified Barriers: lack of disposal guidelines, the absence of return systems, cultural norms, and fear of waste
21	[41]	To determine public attitudes towards medicinal waste and medicines reuse in a large sample of the general population, within a “free prescription medicine” healthcare system, through the use of a web‐based platform where members of the public register to receive health‐related questionnaires	The United Kingdom	Mixed methods	Pharmaceutical	Reverse logistics	Waste disposal	Participants articulated significant concerns regarding the volume of unused and discarded medications Waste is viewed as both a financial and ethical concern A total of 78.7% of respondents indicated a willingness to accept tablets, whereas 75.1% expressed acceptance of capsules from a reuse scheme, contingent upon assurances of safety and quality The main obstacle to the acceptance of reused medicines is the apprehension regarding contamination or tampering Healthcare professionals exhibited greater concern regarding waste and demonstrated less skepticism towards the reuse of medicine compared to the general public There is considerable support for a structured and regulated reuse scheme, particularly given the resource constraints faced by the NHS

Abbreviation: SCM, supply chain management.

**FIGURE 4:**
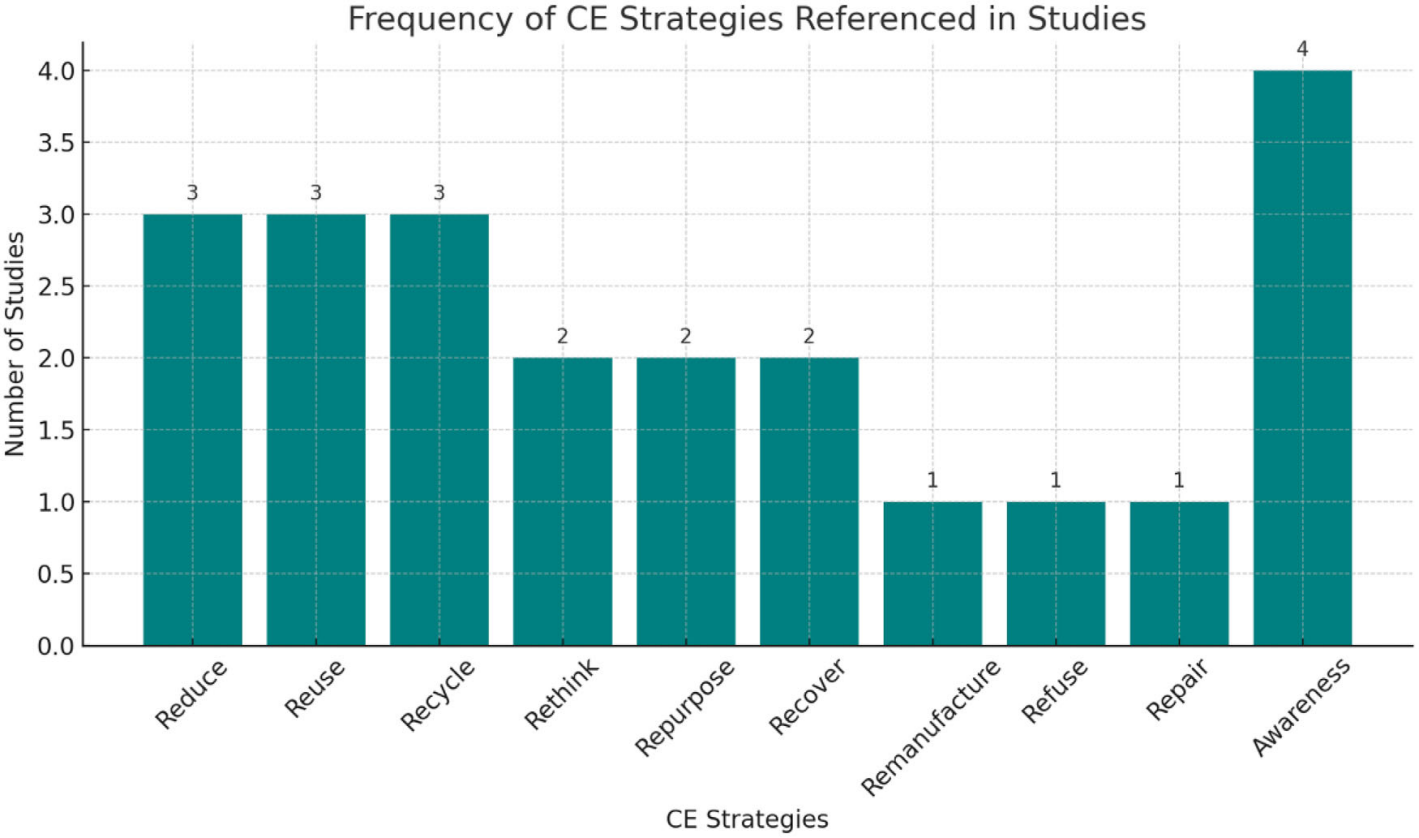
Circular economy (CE) strategies identified.

Various circular economy (CE) strategies were identified across the reviewed studies (Figure 4 and Table 3). The most frequently reported strategies included reduce [23, 29, 31], reuse [26, 37, 41], recycle [26, 34, 40], and awareness [26, 35, 36, 41]. Less frequently mentioned strategies were repurpose [33, 39], rethink [25, 29], refuse [26], repair [38], recover [30, 32], and remanufacture [39]. This distribution highlights the CE practices most emphasized within HCWM research and highlights underexplored areas that warrant further investigation.

**TABLE 3:** Types of circular economy (CE) strategies identified.

CE principle	Description/Application	Studies mentioned
Reduce	Minimizing medicine waste at the source by better procurement forecasting, packaging redesign, and shelf‐life extension (e.g., reducing insulin packaging waste in the United Kingdom and applying 3R principles in Bangladesh)	Alshemari et al. [37], Dihan et al. [40], Sahoo et al. [31]
Reuse	Redistribution or safe re‐dispensing of unused medicines under strict safety checks; patient willingness to reuse tablets/capsules under free‐prescription systems	Alshemari et al. [37], McRae et al. [41]
Rethink	Shifting procurement and prescribing models to minimize overstock (e.g., demand‐driven supply and decentralized purchasing); rethinking community pharmacy roles in managing unused stock	Alshemari et al. [37]
Repurpose	Redirecting unused drugs to charities (Kuwait); reformulating medicines in hospital labs for continued use	Alshemari et al. [37]
Recycle	Recycling of medical packaging and materials; adoption of recyclable medical gowns to reduce CO_2_ and water use	Pellegrini [25], Kaiser et al. [24]
Recover	Energy recovery through incineration or plasma pyrolysis of hazardous pharmaceutical waste; though controversial, still widely practiced	Kaiser et al. [24], Deepak et al. [23]
Remanufacture	Extending product life through remanufacturing (e.g., catheters) to reduce virgin material demand and climate emissions	Meister et al. [39]
Refuse	Avoiding unnecessary procurement and discouraging unsafe practices such as public donation of unused medicines	Dihan et al. [40]; AlAzmi et al. [36]
Repair/Refurbish	Limited evidence in pharmaceutical context; more relevant for medical equipment (e.g., infusion pumps and devices)	Meister et al. [39]
Rethink (awareness)	Educational campaigns to change disposal behaviors; public awareness of environmental impacts of improper disposal	Adedeji‐Adenola et al. [35]; AlAzmi et al. [36]; Mahjoob et al. [26]

## 5 Main Findings

All the records included presented evidence of at least one aspect of the SCM system of HCWM globally. The following are the themes that emerged from the thematic analysis of the included records: policy and regulatory gaps, pharmaceutical supply chain strategies, waste management practices, technology and infrastructure, human resources, training and awareness, economic considerations, and public and patient involvement (Table 4).

**TABLE 4:** Thematic summary of key findings.

Article no.	References	Policy and regulation gaps	Pharmaceutical SCM	Waste management practices	Technology and infrastructure	Human resources, training, and awareness	Economic considerations	Public and patient involvement
1	[37]	✔	✔	✔	✔	✔	✔	✔
2	[27]		✔		✔		✔	
3	[28]	✔	✔	✔	✔	✔	✔	✔
4	[22]		✔		✔	✔		✔
5	[23]			✔		✔		
6	[38]		✔			✔		✔
7	[29]		✔			✔	✔	
8	[24]		✔	✔		✔		
9	[21]			✔	✔			✔
10	[30]			✔				
11	[25]		✔	✔				
12	[31]			✔		✔		
13	[32]			✔		✔	✔	
14	[26]	✔	✔		✔	✔	✔	✔
15	[39]		✔	✔			✔	
16	[33]			✔		✔		
17	[34]	✔		✔				
18	[40]	✔		✔				
19	[35]	✔				✔		✔
20	[36]	✔				✔		✔
21	[41]		✔					✔

### 5.1 Policy and Regulatory Gaps

A total of seven articles reported evidence on policy and regulatory gaps (Table 4) [26, 28, 34, 35, 36, 37, 40]. Numerous studies indicate a persistent obstacle: the absence of unified national and institutional regulations that synchronize supply chain practices with environmental health requirements. These deficiencies hinder the capacity of SCM systems to synchronize logistics, manage inventory expiration, and facilitate reverse flows. In Kuwait, the United Kingdom, Bangladesh, Nigeria, and certain regions of the Middle East, the management of medical waste is impeded by substantial legislative and regulatory deficiencies that obstruct the implementation of CE techniques. Most of these nations lack definitive CE laws, national standards, sustainability criteria, or official protocols for the reuse and disposal of pharmaceuticals. In Kuwait, pharmacies accept returned medications but lack explicit disposal standards, and the absence of the 10Rs framework hinders informed decision‐making [26, 37]. The United Kingdom lacks specific CE policies in healthcare [26]. Bangladesh lacks a CE framework, exhibiting shortcomings in sustainable procurement, material reuse, and manufacturer accountability; nonetheless, the use of 3R strategies: Refuse, Rethink, and Reduce has been proposed [33, 40].

Garbage segregation measures frequently lack adequacy, resulting in the amalgamation of hazardous and general garbage, improper handling of sharps, and disregard for color coding, despite established recommendations [1, 4]. In Nigeria, although the mandate of the National Environmental Standards and Regulations Enforcement Agency (NESREA), there is an absence of a national pharmaceutical waste disposal policy, and current legislation is ineffective and inadequately enforce [35, 42]. In the Health Affairs of Saudi Arabia's Ministry of National Guard, drug return policies are established but lack clarity about disposal procedures [36]. Inadequate legal frameworks, lack of sustainability incentives, and ineffective enforcement result in inconsistent and nonmandatory waste management methods, hindering CE innovation and implementation.

### 5.2 Pharmaceutical Supply Chain Strategies

A total of 11 articles reported evidence on pharmaceutical supply chain strategies (Table 4) [22, 24, 25, 26, 27, 28, 29, 37, 38, 39, 41]. The reduction of medical waste and the implementation of a CE in healthcare are influenced by procurement patterns, logistical coordination, and sustainability plans. In Kuwait, centralized procurement via Central Medical Stores restricts pharmacist involvement, whereas the United Kingdom's decentralized system affords greater autonomy to hospitals and pharmacies [37]. Both nations utilize repackaging, reformulation, and CE strategies, including reduction, reuse, rethinking, and repurposing. Kuwait allocates unused medications to charities and repurposes them in hospital laboratories, whereas the United Kingdom focuses on minimizing packaging waste and extending shelf life [37]. Efficient RL depends on the Coordinated Management and Decision‐Making Collaborative Agent Framework (COORADNDM‐CAF) [28]. This focuses on supplier collaborations, information technology tools, and skilled personnel, facilitating improved inventory oversight, redistribution of surplus goods, and minimized expirations. Key success factors encompass cross‐level collaboration, enhanced expiry alert systems, and explicit role allocation, whereas coordinated supply models improve returns, cost efficiency, corporate social responsibility, and sustainability, incentivized by tax exemptions and grants [1, 4].

Green procurement strategies, life cycle assessments, and the incorporation of environmental criteria into purchasing can transition healthcare towards sustainability, while mitigating overstock challenges stemming from inadequate forecasting and parallel purchases [1, 4, 24]. Green SCM (GSCM) and advanced technologies, such as Big Data, AI, internet of things (IoT), blockchain, and RFID, enhance efficiency, transparency, and waste minimization [43], with innovations such as recyclable medical gowns [24, 25] and the remanufacturing of catheters substantially reducing environmental impact [39]. Nonetheless, the implementation of the CE is impeded by ambiguity in return flows, absence of supply chain designs orientated towards the CE, inflexibility, and suboptimal resource utilization [26]. Public engagement, chemist participation, and clear safety protocols could render medicine re‐dispensing viable and reliable [37, 41].

### 5.3 Waste Management Practices

A total of 13 articles reported evidence on waste management practices (Table 4) [21, 23, 24, 25, 28, 30, 31, 32, 33, 34, 37, 39, 40]. Both Kuwait and the United Kingdom endeavor to recycle or repurpose pharmaceuticals whenever feasible [37]. In Kuwait, repurposing procedures involve altering formulations and utilizing surplus medications for hospitalized patients [37]. In the United Kingdom, home patients’ owned drugs are nonreusable, however unused medications returned from wards are eligible for reuse [37]. Additionally, manufacturers in both nations frequently accept returned pharmaceuticals for resale [37].

Enhanced practices in waste segregation, expiration monitoring, and inventory management, along with collaboration with external waste management entities, ensure the safe and appropriate disposal of pharmaceutical waste [28]. In India, Deepak et al. developed an effectiveness index for biomedical waste (BMW) management across Indian states using a composite indicator approach [23]. The findings revealed significant state‐level disparities in performance. For instance, Delhi demonstrates complete facility coverage (100%) and a high waste collection efficiency of 98.4%; however, limited training initiatives (only 16 workshops conducted) negatively impacted its overall effectiveness. Haryana, on the other hand, faces inadequate connectivity between healthcare facilities (HCFs) and common BMW treatment facilities (CBWTFs), along with insufficient authorization processes, resulting in a lower collection efficiency of 84.0%. In both states, inadequate waste segregation restricts recycling potential, with Delhi recording a recycling rate of just 28.02%. Additionally, high incineration rates contribute significantly to greenhouse gas emissions, with Delhi alone producing 2371.21 MT/year. To address this, plasma pyrolysis is recommended as a more environmentally friendly alternative to traditional incineration due to its reduced emissions [23]. States with index values ranging from 0.05 to 0.09, such as Himachal Pradesh, show gradual improvement yet continue to face several challenges, including limited CBWTF coverage (47.4%), a high proportion of unauthorized healthcare facilities, and frequent regulatory violations arising from weak enforcement mechanisms. Telangana and Punjab have achieved full CBWTF coverage; however, their waste collection remains inadequate due to the presence of numerous unauthorized healthcare facilities. On the other hand, in Bangladesh, only about 33% of healthcare centers had waste segregation facilities, many of which were nonfunctional or improperly used [33, 40]. Hazardous and non‐hazardous waste were often mixed, with no pre‐treatment before disposal. Limited transport options, untrained staff lacking protective gear, and the open dumping of medical waste alongside municipal garbage further heightened environmental and health risks.

Many hospitals continue to rely on incineration for the treatment of infectious medical waste, which remains a preferred practice despite the availability of safer alternatives. However, incineration contributes to environmental pollution and the release of hazardous pollutants [24]. The limited adoption of safer disposal methods, such as ultra‐high‐temperature combustion, can be attributed to several factors: high operational costs, restricted incineration capacity across most LMICs, and weak enforcement of waste disposal regulations, which often results in unregulated dumping and accumulating waste stockpiles [44, 45]. In a study from Pakistan, the only incinerator available in Gujranwala city was nonfunctional, forcing hospitals to rely on a commercial facility located 70 km away [34]. This arrangement led to higher logistical costs and operational challenges for healthcare facilities because they had no alternate treatment methods, including autoclaving and advanced segregation [34].

Although recyclable gowns require more energy for washing, recycling, and transportation, they still have a much lower overall environmental impact than disposable gowns. In fact, recyclable gowns offer significant environmental benefits, including a 65% reduction in energy consumption, a 67% decrease in CO_2_‐equivalent emissions, and an 83% reduction in blue water use and solid waste generation compared to disposables [25]. On the other hand, remanufacturing emerged as a sustainable alternative to the linear take–make–dispose paradigm, particularly for single‐use medical items and can mitigate both raw material usage and high‐emission disposal techniques like as incineration [39].

### 5.4 Technology and Infrastructure

A total of six articles reported evidence on technology and infrastructure (Table 4) [21, 22, 26, 27, 28, 37]. Smart containers, AI tools, RFID, and real‐time tracking systems are seen as possible facilitators. Specifically, RFID devices facilitate the automation of processes including receiving, weighing, storing, inventory management, and waste sorting, minimizes manual errors and operating expenses by enforcing rigorous compliance with disposal rules [43]. For instance, in China, RFID‐enabled systems connect hospitals, transporters, and disposal sites, enabling real‐time monitoring of the entire waste management process and enhancing both efficiency and safety in waste handling [21]. Additionally, RFID technology addresses challenges related to poor data dissemination and limited oversight by enabling prompt response and accountability in cases of mismanagement or violations [21]. This, in turn, helps prevent environmental contamination and the spread of infectious diseases. However, a study from the United Kingdom and Kuwait indicates that 92% and 89% of participants interviewed from these countries, respectively, reported facing considerable technological obstacles that hinder the implementation of effective CE and RL strategies for pharmaceutical waste management [37].

In Brazil, the *Gerenciamento de Medicamentos de Atendimento e Tratamento* (GMAT) system facilitates the tracking of expired medications, stock reassignment, and inventory audits; however, its efficacy is hindered by financial limitations, inadequate internal IT development, and insufficient expiry alarm systems [22, 27, 28]. Inadequate pharmaceutical information systems exacerbate deficiencies in patient returns management, stock visibility, and RL efficiency [28]. Similarly, in the United Kingdom, despite computerized systems, reliability is contingent upon consistent equipment availability, which is varied [38]. Overall, major CE challenges include limited eco‐friendly disposal options, poor product traceability, lack of sustainability metrics, minimal use of medical informatics, and complex operations hindered by inadequate technological and data infrastructure for sustainable HCWM [26].

### 5.5 Human Resources, Training, and Awareness

A total of 13 articles reported evidence on human resources, training, and awareness (Table 4) [22, 23, 24, 26, 27, 28, 29, 31, 32, 33, 35, 36, 38]. In the United Kingdom, insufficient staffing and limited knowledge of CE among stakeholders were identified as key obstacles in medical waste management, whereas overall awareness of CE principles among chemists and patients in both the United Kingdom and Kuwait remains low [37]. The study recommended that stakeholders undergo specialized training programs to enhance their understanding of the economic and environmental benefits of CE practices. In Brazil, the implementation of RL projects is hampered by limited technical expertise, weak managerial capacity, and a shortage of personnel to manage both direct and RL operations [28]. As a result, outsourcing has emerged as a practical solution for handling RL activities.

Distributors’ corporate social responsibility initiatives concentrate on enhancing public knowledge on the environmental hazards associated with incorrect antibiotic disposal. These initiatives help increase return volumes, reduce the number of expired antibiotics, lower production costs, and promote environmental conservation as well as the proper public health classification and management of returned antibiotics [29]. Moreover, few hospitals employ specialized environmental or waste managers, even though the healthcare sector generates complex and diverse waste streams [24]. Most regular healthcare workers lack sufficient understanding of the environmental impacts of medical products and services; thus, collaboration with environmental specialists is vital to foster effective interdisciplinary teamwork. Engaging product manufacturers, distributors, and waste processors in this effort is therefore crucial to eliminate persistent environmental pollutants [24].

In a study from Bangladesh, more than 85% of waste segregation personnel lacked training [33]. Moreover, only 10% of healthcare providers received basic training (1–3 days) before starting work, and many demonstrated poor awareness and compliance with the MWM Act of 2008, with training records also poorly maintained. Similarly, in a study from Nigeria, about 78.3% of patients did not receive advice from healthcare professionals on pharmaceutical waste handling [35]. The study further identified insufficient public education and inadequate involvement from chemists and physicians, despite their essential role in the proper disposal of pharmaceutical waste. In another study from Saudi Arabia, a lack of knowledge and awareness led patients to adopt unsafe disposal practices, such as flushing medications down the toilet (55.2%), discarding them in household garbage (63.5%), or burning them (44.8%) [36]. Pharmacists and healthcare personnel are inadequately utilized in patient education during drug counseling [36].

Training and awareness creation serve as critical trade‐off indicators that influence various other performance domains in waste management [23]. In India, Deepak et al. recommend that states strengthen training, enforcement, and the use of technologies such as GPS, barcoding, and OEMS to reduce violations and emissions while enhancing recycling and collection efficiency [23]. Practically, in a similar study in India, training sessions heightened healthcare personnel’ awareness regarding the hazards of incorrect BMW practices, especially needle stick accidents [31]. Additionally, a robust sense of responsibility on the personnel was cultivated through systematic training. The sanitation and housekeeping personnel exhibited the most significant enhancement, achieving performance levels comparable to physicians in segregation practices following training. Female healthcare workers demonstrated greater vigilance regarding BMW dangers and safety standards. Initially, there were gaps in understanding proper segregation practices, and knowledge of color‐coded waste segregation was limited [31]. Moreover, a hybrid decision approach to consistently train personnel at waste management facilities improves the efficiency of RL and facilitates prompt trash transfer from medical facilities to disposal sites [32].

### 5.6 Economic Considerations

A total of seven articles reported evidence on economic considerations (Table 4) [26, 27, 28, 29, 32, 37, 39]. In the United Kingdom and Kuwait, concerns regarding cost‐effectiveness hinder the adoption of CE principles by manufacturers and healthcare systems [37]. Pharmacists in the United Kingdom consider cost in their procurement decisions; nonetheless, the need for additional staff may elevate operational expenses. However, neither the United Kingdom nor Kuwait has established formal medication donation programs [37]. Adequate funding is essential to enable investment in IT systems, outsourced services, and improved storage and segregation facilities, which together strengthen infrastructure and enhance the efficiency of medication management [27]. Resource scarcity within the public pharmaceutical care process (PCP) cycle limits overall efficiency, as funding is primarily directed towards forward logistics, leaving RL underfunded [28]. Additionally, corruption and bureaucratic inefficiencies further hinder effective financial management and the successful implementation of RL initiatives [28].

Centralized systems encourage distributors to improve their corporate social responsibility by increasing the return of unwanted antibiotics, thereby reducing the risk of government penalties [29]. However, this approach raises operational costs compared to decentralized models. To offset these expenses and promote cooperation, manufacturers introduce an incentive mechanism, offering $2.20 per returned unit, which fosters centralized and collaborative decision‐making among distributors.

Healthcare institutions face several challenges, including suboptimal technological eco‐efficiency, interdepartmental conflicts and poor communication, high costs associated with CE waste disposal, reluctance to invest in CE initiatives, inadequate funding allocation, and misaligned budgets [26]. To address these challenges, healthcare organizations and regulators should invest in remanufacturing infrastructure and adopt procurement decisions guided by life cycle assessments [24, 39]. Additionally, they should advocate for realistic, long‐term sustainability metrics and expand emission assessments to cover additional high‐waste medical devices. Overall, they can maintain safety while managing costs by analyzing trade‐offs (opportunity cost), partnering with financially stable firms for discounts, reducing upfront technology costs, and taking advantage of economies of scale to cut long‐term waste and expenses [32].

### 5.7 Public and Patient Involvement

A total of six articles reported evidence on public and patient involvement (Table 4) [26, 29, 32, 35, 36, 41]. The adoption of a CE in medical waste management faces significant operational, societal, and manufacturer‐related challenges, with public awareness and engagement emerging as critical factors [26]. Societal and cultural barriers, such as limited consumer awareness, preference for single‐use products, and a lack of incentives, highlight the need for behavioral and attitudinal change. Public participation plays a vital role in achieving sustainable outcomes; however, poor disposal practices remain widespread. For instance, in Nigeria, 73.2% of individuals dispose of medicines in household garbage and 19.3% flush them down toilets or sinks, even though 85.3% acknowledge the associated risks [35]. Similarly, in Saudi Arabia, cytotoxic drugs are frequently discarded with general waste, and many people mistakenly view medicine donation as a safe disposal option despite health risks [36]. Interestingly, public willingness to reuse medicines is relatively high (78.7% for tablets and 75.1% for capsules), but persistent concerns about safety and quality continue to limit practical implementation [41]. These findings highlight the importance of sustained public sensitization, community engagement, and education to foster responsible participation in CE practices.

On the other hand, reinforcement and the active involvement of authorities play a major role in ensuring public compliance with safe medical waste disposal practices and adherence to pharmaceutical return policies. Strengthening regulatory enforcement, conducting regular monitoring, and imposing appropriate penalties for noncompliance can further enhance accountability [29, 32].

### 5.8 Integrated Pathways Linking SCM Components to HCWM and Environmental Outcomes

Table 5 provides an integrated overview of how key SCM components influence HCWM practices and environmental health outcomes. The table synthesizes evidence across the review by illustrating the pathways through which inventory management, RL, regulatory mechanisms, digital SCM technologies, and CE approaches contribute to waste prevention, improved resource utilization, regulatory compliance, and environmental sustainability. It highlights that SCM components do not operate independently but function as interconnected mechanisms, where effective inventory control reduces waste generation, RL supports recovery and safe disposal, and regulatory and digital systems provide the enabling environment required for sustainable implementation.

**TABLE 5:** Integrated framework illustrating the role of supply chain management components in healthcare waste management and environmental health outcomes.

SCM component	Mechanism	HCWM effect	Environmental outcome	Enabling factors/barriers
Inventory control	Forecasting, stock rotation, expiry monitoring	Reduces expired/unused products	Lower waste generation	Data quality, procurement practices
Reverse logistics	Return, redistribution, recovery pathways	Improves waste recovery	Reduced disposal burden, resource recovery	Regulation, supplier coordination
Regulatory mechanisms	Standards, monitoring, enforcement	Improves compliance	Safer disposal practices	Governance capacity
Digital SCM	RFID, IoT, tracking systems	Improves visibility and traceability	Better waste monitoring	Infrastructure, cost

Abbreviations: HCWM, healthcare waste management; IoT, internet of things; RFID, radio frequency identification; SCM, supply chain management.

## 6 Discussion

The findings of this study indicate that SCM plays a central and cross‐cutting role in HCWM by shaping how medical products are procured, stored, used, returned, and ultimately disposed of within health systems. Across the included studies, SCM was shown to influence both waste prevention through inventory control and waste mitigation through RL mechanisms. Effective SCM practices, including improved demand forecasting, stock rotation, and expiry monitoring, were associated with reduced accumulation of expired and unused medical products, whereas well‐structured RL systems facilitated the return, redistribution, repurposing, or safe disposal of healthcare waste. Beyond the findings of individual studies, this synthesis demonstrates how RL, inventory control, and regulatory compliance operate as interconnected components of a broader SCM framework, collectively influencing HCWM performance and environmental health outcomes. By integrating evidence across diverse healthcare settings and geographical contexts, the review identifies common implementation challenges, recurring policy gaps, and system‐wide opportunities that are not readily apparent from isolated studies or reviews focused on single SCM functions. However, the findings also highlight persistent systemic weaknesses, particularly in policy and regulatory alignment, infrastructure, technology, human resource capacity, and stakeholder coordination, that undermine the efficiency of SCM systems and contribute to improper waste handling and adverse environmental health impacts. Overall, the evidence suggests that strengthening SCM systems is fundamental to improving HCWM outcomes and advancing sustainable and CE approaches in healthcare delivery.

Specifically, the findings of this review reveal major regulatory and policy gaps that impede CE adoption in medical waste management. Across countries such as Kuwait, the United Kingdom, Bangladesh, Nigeria, and Saudi Arabia, there is a lack of unified national or institutional frameworks, clear CE laws, sustainability standards, and enforcement mechanisms [26, 35, 37, 40]. Existing regulations on waste management in these countries are fragmented, poorly implemented, or absent, leading to inconsistent waste segregation, ineffective pharmaceutical disposal, and limited manufacturer accountability. These gaps transcend geographical and sociocultural boundaries, affecting both high‐income and LMICs. These systemic deficiencies weaken supply chain coordination, obstruct RL, and delay progress towards sustainable healthcare waste practices. The policy and regulatory gaps identified across these countries reflect a lack of alignment with WHO's guidance on Climate‐Resilient and Environmentally Sustainable HCFs [46], which emphasizes resilience, cross‐sector collaboration, and sustainable resource use to strengthen environmental and health system sustainability. Likewise, these gaps deviate from the WHO's “Greener Pharmaceuticals Regulatory Highway” initiative [47], which advocates for sustainable medical production and distribution, enhanced digital regulatory capacity, particularly in LMICs, and early regulator–manufacturer collaboration to minimize medical waste. Strengthening national waste policies and integrating CE principles into healthcare regulations could establish stronger governance for sustainability and reduce environmental risks from the health sector [13]. However, the implementation of reuse and RL systems must also address potential ethical risks, including compromised product safety, inadequate quality assurance, inappropriate redistribution of expired or unsuitable materials, and inequitable access. Therefore, robust regulatory oversight, quality assurance processes, and traceability mechanisms are essential to ensure that sustainability efforts do not compromise patient safety or healthcare equity.

Evidence from the included studies highlights that effective pharmaceutical supply chain strategies are central to advancing CE practices in healthcare. Sustainable outcomes depend on efficient procurement, logistics coordination, and integration of green practices [43]. Although the United Kingdom's decentralized system promotes autonomy and reuse strategies, Kuwait's centralized model limits pharmacist involvement [37]. The United Kingdom's strategy appears to work better and promotes medicine returns more effectively, as pharmacists at the facility level play an active role in coordinating RL, monitoring expiries, and ensuring compliance with green procurement policies. This decentralized approach fosters accountability, innovation, and collaboration between suppliers and healthcare facilities, resulting in higher recovery rates and reduced pharmaceutical waste. Successful pharmaceutical supply chain strategies established in this review include supplier collaboration, advanced expiry tracking, and incentives like tax exemptions [22, 24, 25, 26, 27, 28, 29, 37, 38, 39, 41]. Most advanced countries, like the United Kingdom and China, use green procurement, life cycle assessments, and technologies such as AI, IoT, and blockchain enhance transparency and reduce waste [21, 37]. However, CE adoption remains constrained by unclear return policies, rigid supply systems, weak stakeholder engagement, and the absence of supply chain designs tailored to circular principles, especially LMICs. This geographical disparity may be attributed to weak regulatory frameworks, limited financing, poor infrastructure, and low technical capacity in the region unlike higher income countries [48].

Incineration continues to dominate waste management practices in many healthcare systems, despite its environmental impact [24]. Although incineration is often considered a convenient and cost‐effective means of reducing waste volume and destroying infectious materials, it releases toxic pollutants such as dioxins, furans, and heavy metals into the environment, contributing to air pollution and long‐term ecological damage [49]. Although alternative waste management methods, such as plasma pyrolysis, autoclaving, and remanufacturing, offer cleaner and more sustainable solutions, their adoption is restricted by high implementation costs, limited technical capacity, and institutional inertia [39]. Moreover, waste segregation deficiencies, restricted transport capacity, and inadequate recycling rates continue to exist, even in areas with extensive treatment facility coverage [23, 33].

Technology and infrastructure play a crucial role in enabling CE practices in pharmaceutical waste management. Tools such as RFID, AI, and smart tracking systems improve automation, traceability, and compliance, as seen in China's RFID‐enabled waste monitoring network [21]. However, widespread implementation is restricted by technological barriers, financial constraints, weak IT systems, and unreliable equipment, as reported in countries like the United Kingdom, Kuwait, and Brazil [27, 28, 37]. Poor data integration, limited sustainability metrics, and inadequate digital infrastructure continue to undermine effective RL, stock visibility, and environmentally responsible waste handling in healthcare systems. These technological and data‐related gaps are not unique to any one region. Both high‐income countries and LMICs have demonstrated challenges in deploying digital tools for CE practices in medical waste management. Even in technologically advanced settings like the United Kingdom, healthcare facilities report difficulties in integrating digital platforms and maintaining real‐time monitoring systems due to interoperability and infrastructure challenges [37]. However, the situation is markedly more severe in LMICs, where weak digital infrastructure, inconsistent power supply, inadequate funding, and a shortage of skilled personnel exacerbate implementation barriers [50]. Consequently, although advanced technologies have shown promise in improving traceability and sustainability in medical waste management, their full potential remains unrealized in most contexts. This reinforces the need for policy investment, capacity building, and system‐level digital transformation to support CE adoption across all health systems.

This review established that inadequate human resources, limited technical expertise, and poor awareness significantly impede the adoption of CE strategies in HCWM. Across countries, such as the United Kingdom, Kuwait, Brazil, Bangladesh, Nigeria, and Saudi Arabia, low CE literacy, insufficient training, and staff shortages, reduce compliance and efficiency in waste handling [22, 23, 24, 26, 27, 28, 29, 31, 32, 33, 35, 36]. Few institutions employ environmental specialists, and most healthcare workers lack knowledge of sustainable practices [24]. Targeted training programs, interdisciplinary collaboration, and public education are essential to improve segregation practices, strengthen RL, and promote environmentally responsible disposal. Effective training has been shown to enhance awareness, accountability, and safety, making it a key driver of sustainable waste management [23].

Economic constraints significantly impact all facets of CE implementation. High initial capital expenditures, inadequate funding for RL, and competing budgetary priorities delay progress, particularly in LMICs [26, 28, 51]. Incentive mechanisms, including tax exemptions and corporate social responsibility initiatives led by manufacturers, have shown potential to improve medicine returns, promote sustainability, and align stakeholder incentives [29]. The policy implication of these findings align closely with the WHO's Global Strategy on Health, Environment, and Climate Change, which advocates for integrating sustainability into health sector financing [13]. Strengthening green financing policies, offering subsidies for CE technologies, and integrating sustainability indicators into health budgets are key to building environmentally responsible and economically resilient healthcare systems.

Evidence shows that limited public and patient involvement pose a major barrier to implementing CE practices in medical waste management. Low awareness, cultural preferences for single‐use products, and inadequate incentives contribute to unsafe disposal habits, such as discarding or flushing medicines [35, 36]. Although many people express willingness to reuse medicines, safety and quality concerns prevent this practice [41]. Effective CE adoption, therefore, requires continuous public education, community engagement, and stricter enforcement of pharmaceutical return and disposal policies to promote responsible and sustainable waste management behaviors.

The findings of this review also highlight important debates within the SCM and HCWM literature. Although conventional SCM approaches emphasize efficiency, cost reduction, inventory optimization, and uninterrupted supply availability, HCWM literature has increasingly argued that these priorities must be balanced with environmental sustainability, regulatory compliance, and public health protection. WHO emphasizes that effective HCWM requires integrated systems that address waste minimization, segregation, regulation, resource allocation, and environmentally sound disposal practices rather than focusing solely on downstream waste treatment [52]. For example, strategies that minimize excess inventory may reduce waste generation; however, overly aggressive inventory reduction approaches may increase the risk of stock‐outs and compromise healthcare service delivery. Similarly, although RL is increasingly recognized as a mechanism for improving resource recovery, sustainability, and circularity within healthcare supply chains, its implementation remains dependent on supportive regulatory frameworks, infrastructure, economic feasibility, and coordination among manufacturers, suppliers, healthcare facilities, and waste management providers. Recent global guidance also highlights the importance of RL and EPR approaches for improving pharmaceutical waste management and promoting sustainable healthcare supply chains [53]. The literature also presents differing perspectives on the role of digital technologies, with some studies highlighting their potential to improve traceability and decision‐making, whereas others emphasize challenges related to cost, infrastructure, interoperability, and implementation capacity, particularly in resource‐constrained settings. By synthesizing these perspectives, this review demonstrates that sustainable HCWM requires a balanced SCM approach that integrates operational efficiency with environmental and health system priorities rather than treating waste management as a downstream disposal challenge.

## 7 Practical Implications and Recommendations

The findings of this review have important implications for strengthening HCWM through improved SCM practices and CE‐aligned approaches. The evidence demonstrates that sustainable HCWM requires coordinated action across healthcare facilities, policymakers, researchers, and communities. The key implications are outlined below.

### 7.1 Implications for Healthcare Practice

Healthcare facilities can achieve immediate improvements in waste reduction and resource efficiency by strengthening core SCM functions, including demand forecasting, procurement planning, inventory control, stock rotation, and expiry monitoring. Hospitals, laboratories, and pharmacies should institutionalize standardized inventory management systems and accountability mechanisms to minimize overstocking, reduce expired and unused medical products, and improve resource utilization.

Structured RL systems should also be integrated into routine healthcare operations to enable the safe return, redistribution, repurposing, recycling, or appropriate disposal of unused pharmaceuticals and medical supplies. Such systems can reduce avoidable waste, improve resource recovery, and strengthen coordination between healthcare facilities, suppliers, and waste management providers.

The review further highlights the importance of digital transformation in healthcare supply chains. Technologies, such as RFID systems, digital inventory platforms, and automated tracking tools, can improve product traceability, inventory visibility, waste monitoring, and regulatory compliance. However, successful implementation requires investment in infrastructure, interoperability, technical capacity, and sustained institutional support, particularly in resource‐constrained settings.

Building workforce capacity is equally critical. Continuous training of healthcare workers, pharmacists, and supply chain personnel on sustainable procurement, inventory management, waste segregation, RL, and regulatory compliance should be embedded within routine professional development frameworks. Strengthening these competencies will support safer waste handling practices and improve overall system performance.

### 7.2 Implications for Policy and Health System Governance

Policymakers and regulatory authorities should strengthen healthcare waste governance by integrating SCM principles into national HCWM policies, procurement frameworks, and sustainability strategies. Clear regulatory guidance is needed to support RL pathways, EPR approaches, digital tracking systems, and environmentally responsible disposal practices.

Investment in enabling infrastructure, regulatory enforcement mechanisms, and stakeholder coordination is essential to ensure that SCM improvements translate into sustainable HCWM outcomes. Policies should also encourage CE approaches by promoting waste prevention, resource recovery, and lifecycle management of healthcare products.

From an economic perspective, strengthening SCM systems may provide opportunities for cost savings through reduced procurement of excess stock, lower waste disposal costs, improved resource recovery, and more efficient use of healthcare resources. Although available studies did not consistently quantify these benefits, the evidence supports the development of stronger economic cases for investment in sustainable SCM interventions.

### 7.3 Implications for Research and Evidence Generation

The review identifies important evidence gaps that require further investigation to strengthen the evidence base for sustainable HCWM. Future research should prioritize evaluating the real‐world impacts, implementation effectiveness, and long‐term sustainability of national CE and SCM policies across diverse healthcare settings. More longitudinal and implementation‐focused studies are needed to better understand how SCM interventions, including digital supply chain solutions, RL models, and CE‐based approaches, influence healthcare waste outcomes over time, particularly in resource‐constrained settings.

Future studies should also examine the scalability, feasibility, and sustainability of emerging technologies, including IoT‐enabled monitoring, RFID systems, and digital traceability platforms, across different healthcare system contexts. Standardized outcome measures should be developed and adopted to improve comparability between studies and enable quantitative synthesis or meta‐analysis where sufficient homogeneous evidence becomes available.

Further research is needed to explore cultural and behavioral factors influencing medicine reuse, return, and disposal practices to inform effective public engagement and awareness strategies. Economic evaluations and modeling studies should also be prioritized to quantify the long‐term cost savings, resource efficiencies, and environmental benefits associated with CE‐aligned SCM interventions, providing policymakers with stronger evidence for investment decisions and policy formulation.

Comparative studies evaluating centralized and decentralized procurement systems, as well as different SCM models, are essential for identifying context‐specific best practices that enhance waste reduction, resource efficiency, and sustainability. Generating more robust and quantifiable evidence through these approaches will support the development of scalable, evidence‐informed HCWM systems and strengthen the potential for future meta‐analytic assessments of SCM interventions.

### 7.4 Implications for Public and Stakeholder Engagement

Public and patient engagement is essential for improving downstream HCWM, particularly pharmaceutical disposal practices. Health systems should implement targeted awareness campaigns and establish accessible medicine return schemes to reduce unsafe disposal behaviors, such as household discarding or inappropriate disposal of medicines.

Engaging communities, suppliers, healthcare workers, and waste management providers in shared responsibility for HCWM can improve compliance, strengthen RL pathways, and promote environmentally responsible behaviors.

### 7.5 Implications for Education and Workforce Development

The findings of this review highlight the need to strengthen education and training programs for healthcare professionals and supply chain managers by integrating HCWM, sustainable supply chain principles, and CE approaches into existing curricula. Training programs for healthcare workers, pharmacists, laboratory personnel, and supply chain professionals should incorporate competencies in sustainable procurement, inventory optimization, expiry monitoring, RL, waste segregation, regulatory compliance, and digital SCM.

For supply chain managers, curricula should emphasize the role of data‐driven forecasting, stock management systems, product lifecycle approaches, and supplier coordination in reducing healthcare waste and improving resource efficiency. Similarly, healthcare professional training should address the environmental and public health consequences of inappropriate waste handling and promote responsible practices across the healthcare product lifecycle.

Integrating these competencies into undergraduate education, postgraduate training, and continuing professional development programs can strengthen workforce capacity and support the implementation of sustainable HCWM systems. Such educational reforms are particularly important in resource‐limited settings, where strengthening human capacity is essential for translating SCM policies and CE strategies into effective practice.

## 8 Strengths and Limitations

The reviewed evidence demonstrates notable strengths, including the use of rigorous evidence synthesis methods and coverage of diverse geographic and economic contexts, spanning high‐, middle‐, and low‐income settings, which allows meaningful cross‐comparisons of barriers and facilitators to CE adoption. The inclusion of multiple stakeholder perspectives, such as policymakers, pharmacists, and patients, also provides a holistic understanding of the challenges influencing CE implementation. However, several limitations are evident. The heterogeneity of included study designs, including qualitative studies, cross‐sectional surveys, case studies, implementation evaluations, and policy analyses, influenced the strength and generalizability of the evidence. Although this methodological diversity enabled exploration of SCM practices across different contexts, it limited direct comparison between studies and prevented quantitative synthesis of outcomes. The reliance on self‐reported data in some studies introduces potential recall and social desirability bias, whereas the predominance of cross‐sectional designs limits causal inference regarding the relationship between SCM interventions and healthcare waste outcomes. Furthermore, the limited availability of longitudinal evaluations constrains understanding of the long‐term sustainability and effectiveness of SCM and CE‐based interventions.

Variations in study findings also highlight important contextual differences. Although most studies reported that improved inventory management, RL, regulatory mechanisms, and digital supply chain solutions contribute to improved HCWM outcomes, differences were observed regarding their feasibility, scalability, and implementation effectiveness. For example, although digital SCM technologies were shown to enhance traceability, inventory visibility, and decision‐making in some settings, other studies identified barriers related to infrastructure, cost, interoperability, and technical capacity. Similarly, regulatory frameworks were generally recognized as essential for improving HCWM practices; however, their effectiveness depended on enforcement mechanisms, institutional capacity, and availability of resources. These discrepancies indicate that SCM interventions require adaptation to local health system structures, governance arrangements, and resource capacities rather than universal application.

In addition, many included studies were highly context‐specific, which may limit the transferability of findings across different healthcare systems. A further limitation of this scoping review is that grey literature searches were restricted to key sources, including WHO and selected national health department websites; however, no grey literature met the eligibility criteria for inclusion. Although this approach ensured consistency, transparency, and adherence to the predefined systematic search strategy, it may have excluded additional country‐level or locally generated grey literature that could provide further contextual insights into healthcare SCM and HCWM practices. Future reviews could therefore broaden grey literature searches and incorporate more longitudinal and implementation‐focused studies to strengthen understanding of SCM interventions and their long‐term impacts on sustainable HCWM.

## 9 Conclusion

The evidence suggests that supply chain processes, particularly procurement planning, inventory management, logistics systems, expiry monitoring, and RL practices, may influence healthcare waste generation, waste handling practices, product traceability, and broader environmental sustainability efforts. Importantly, SCM components do not operate independently but function as interconnected mechanisms within healthcare systems. Effective inventory control through improved forecasting, stock rotation, and expiry monitoring can reduce avoidable waste generation, whereas RL systems facilitate the recovery, redistribution, repurposing, or safe disposal of unused and expired healthcare products. These processes are further supported by regulatory frameworks and digital systems that provide the governance, monitoring, and traceability mechanisms required for sustainable implementation. Across the included studies, inefficient supply chain practices were frequently associated with increased waste generation, product wastage, and challenges in the safe and sustainable management of healthcare waste. The review also identifies several persistent barriers that may limit the effective integration of SCM and sustainable waste management practices, including weak policy and regulatory frameworks, inadequate infrastructure, limited technological capacity, insufficient workforce training, and poor coordination among stakeholders. Collectively, these findings suggest that strengthening healthcare supply chain systems could support more efficient resource utilization, improve waste management practices, and enhance environmental sustainability across healthcare settings. More broadly, the evidence indicates that greater attention to integrated supply chain processes may help advance CE approaches in healthcare while supporting broader sustainability goals, including improved health system efficiency and environmentally responsible healthcare service delivery.

## Author Contributions

Conceptualization: **Sikhanyiselwe Nkomo**, **Maasago Mercy Sepadi**, and **Kuhlula Maluleke**. Methodology: Sikhanyiselwe Nkomo, Maasago Mercy Sepadi, **Kabelo Kgarosi**, **Evans Duah**, and Kuhlula Maluleke. Data curation: Kabelo Kgarosi. Formal analysis: Sikhanyiselwe Nkomo and Kabelo Kgarosi. Writing – original draft preparation: Sikhanyiselwe Nkomo and Evans Duah. Writing – review and editing: Maasago Mercy Sepadi, Evans Duah, and Kuhlula Maluleke.

## Funding

The authors have nothing to report.

## Ethics Statement

The authors have nothing to report.

## Consent

The authors have nothing to report.

## Conflicts of Interest

The authors declare no conflicts of interest.

## Supporting information




**Supporting File**: puh270386‐sup‐0001‐SuppMat.docx.

## Data Availability

This study's generated and analyzed data will be presented and published as part of the systematic scoping review.
