# Supplementary material for: The Role of Supply Chain Management in Healthcare Waste Management: A Scoping Review on Reverse Logistics, Inventory Control, and Environmental Health Impacts
Source: Public Health Chall. 2026 Sep 23;5(3):e70386. doi: 10.1002/puh2.70386 (PMC13600725; doi:10.1002/puh2.70386)
Supplement: Supplementary file 1 [file PUH2-5-e70386-s001.docx]

**Appendix 1: Search strategy**

| Date | Database | Search strategy | Results |
| --- | --- | --- | --- |
| 14 May 2025 | Scopus | ( TITLE-ABS-KEY ( "Supply Chain Management" OR scm OR "healthcare logistics" OR "medical supply chain" OR "healthcare logistics" OR "pharmaceutical logistics" OR "inventory control" OR "inventory management" ) AND TITLE-ABS-KEY ( "Waste Management" OR "Medical Waste" OR "healthcare waste" OR "biohazard waste" OR "Hazardous Waste Sites" OR "infectious waste" OR "Medical Waste Disposal" OR "hazardous medical waste" OR "waste disposal" OR "Refuse Disposal" OR "Waste Disposal, Fluid" OR "Medical Waste Disposal" OR "expired drugs" OR "expired medicines" OR "pharmaceutical waste" OR "Environmental Health" OR "waste segregation" OR "waste recycling" OR "sharps disposal" OR "solid waste management" ) AND TITLE-ABS-KEY ( "regulatory compliance" OR "health regulations" OR "environmental health impact" OR "environmental contamination" OR "public health risk" OR "infection control" OR "antimicrobial resistance" OR pollution ) ) | 172 |
| 14 May 2025 | Pubmed | (("Supply Chain Management" OR SCM OR "healthcare logistics" OR "medical supply chain" OR "healthcare logistics" OR "pharmaceutical logistics" OR "inventory control" OR "inventory management") AND ("Waste Management"[Mesh] OR "Waste Management" OR "Medical Waste"[Mesh] OR "Medical Waste" OR "healthcare waste" OR "biohazard waste" OR "Hazardous Waste Sites"[Mesh] OR "infectious waste" OR "Medical Waste Disposal"[Mesh] OR "hazardous medical waste" OR "waste disposal" OR "Refuse Disposal"[Mesh] OR "Waste Disposal, Fluid"[Mesh] OR "Medical Waste Disposal"[Mesh] OR "expired drugs" OR "expired medicines" OR "pharmaceutical waste" OR "Environmental Health"[Mesh] OR "Environmental Health" OR "waste segregation" OR "waste recycling" OR "sharps disposal" OR "solid waste management")) AND ("regulatory compliance" OR "health regulations" OR "environmental health impact" OR "environmental contamination" OR "public health risk" OR "infection control" OR "antimicrobial resistance" OR pollution) | 48 |
| 14 May 2025 | Web of Sci | ((ALL=("Supply Chain Management" OR scm OR "healthcare logistics" OR "medical supply chain" OR "healthcare logistics" OR "pharmaceutical logistics" OR "inventory control" OR "inventory management" )) AND ALL=("Waste Management" OR "Medical Waste" OR "healthcare waste" OR "biohazard waste" OR "Hazardous Waste Sites" OR "infectious waste" OR "Medical Waste Disposal" OR "hazardous medical waste" OR "waste disposal" OR "Refuse Disposal" OR "Waste Disposal, Fluid" OR "Medical Waste Disposal" OR "expired drugs" OR "expired medicines" OR "pharmaceutical waste" OR "Environmental Health" OR "waste segregation" OR "waste recycling" OR "sharps disposal" OR "solid waste management" )) AND ALL=("regulatory compliance" OR "health regulations" OR "environmental health impact" OR "environmental contamination" OR "public health risk" OR "infection control" OR "antimicrobial resistance" OR pollution ) | 72 |
|  | EBSCOhost (Academic Search Complete) | ("Supply Chain Management" OR scm OR "healthcare logistics" OR "medical supply chain" OR "healthcare logistics" OR "pharmaceutical logistics" OR "inventory control" OR "inventory management") AND ("Waste Management" OR "Medical Waste" OR "healthcare waste" OR "biohazard waste" OR "Hazardous Waste Sites" OR "infectious waste" OR "Medical Waste Disposal" OR "hazardous medical waste" OR "waste disposal" OR "Refuse Disposal" OR "Waste Disposal, Fluid" OR "Medical Waste Disposal" OR "expired drugs" OR "expired medicines" OR "pharmaceutical waste" OR "Environmental Health" OR "waste segregation" OR "waste recycling" OR "sharps disposal" OR "solid waste management") AND ("regulatory compliance" OR "health regulations" OR "environmental health impact" OR "environmental contamination" OR "public health risk" OR "infection control" OR "antimicrobial resistance" OR pollution) | 49 |
|  | EBSCOhost (Business Source Complete) | ("Supply Chain Management" OR scm OR "healthcare logistics" OR "medical supply chain" OR "healthcare logistics" OR "pharmaceutical logistics" OR "inventory control" OR "inventory management") AND ("Waste Management" OR "Medical Waste" OR "healthcare waste" OR "biohazard waste" OR "Hazardous Waste Sites" OR "infectious waste" OR "Medical Waste Disposal" OR "hazardous medical waste" OR "waste disposal" OR "Refuse Disposal" OR "Waste Disposal, Fluid" OR "Medical Waste Disposal" OR "expired drugs" OR "expired medicines" OR "pharmaceutical waste" OR "Environmental Health" OR "waste segregation" OR "waste recycling" OR "sharps disposal" OR "solid waste management") AND ("regulatory compliance" OR "health regulations" OR "environmental health impact" OR "environmental contamination" OR "public health risk" OR "infection control" OR "antimicrobial resistance" OR pollution) | 62 |
|  | EBSCOhost (Economic Literature) | ("Supply Chain Management" OR scm OR "healthcare logistics" OR "medical supply chain" OR "healthcare logistics" OR "pharmaceutical logistics" OR "inventory control" OR "inventory management") AND ("Waste Management" OR "Medical Waste" OR "healthcare waste" OR "biohazard waste" OR "Hazardous Waste Sites" OR "infectious waste" OR "Medical Waste Disposal" OR "hazardous medical waste" OR "waste disposal" OR "Refuse Disposal" OR "Waste Disposal, Fluid" OR "Medical Waste Disposal" OR "expired drugs" OR "expired medicines" OR "pharmaceutical waste" OR "Environmental Health" OR "waste segregation" OR "waste recycling" OR "sharps disposal" OR "solid waste management") AND ("regulatory compliance" OR "health regulations" OR "environmental health impact" OR "environmental contamination" OR "public health risk" OR "infection control" OR "antimicrobial resistance" OR pollution) | 57 |
|  | EBSCOhost (MEDLINE) | ("Supply Chain Management" OR scm OR "healthcare logistics" OR "medical supply chain" OR "healthcare logistics" OR "pharmaceutical logistics" OR "inventory control" OR "inventory management") AND ("Waste Management" OR "Medical Waste" OR "healthcare waste" OR "biohazard waste" OR "Hazardous Waste Sites" OR "infectious waste" OR "Medical Waste Disposal" OR "hazardous medical waste" OR "waste disposal" OR "Refuse Disposal" OR "Waste Disposal, Fluid" OR "Medical Waste Disposal" OR "expired drugs" OR "expired medicines" OR "pharmaceutical waste" OR "Environmental Health" OR "waste segregation" OR "waste recycling" OR "sharps disposal" OR "solid waste management") AND ("regulatory compliance" OR "health regulations" OR "environmental health impact" OR "environmental contamination" OR "public health risk" OR "infection control" OR "antimicrobial resistance" OR pollution) | 38 |

**Appendix 2: Inter-rater agreement**

| **Author & Date** | **Reviewer 1 (SN)** | **Reviewer 2 (KM)** |
| --- | --- | --- |
| Adedeji-Adenola 2022 | 1 | 1 |
| Alamzi 2017 | 1 | 1 |
| Ali 2016 | 1 | 1 |
| Alshemari 2025 | 1 | 1 |
| Campos 2020 | 1 | 1 |
| Campos 2021 | 1 | 1 |
| Campos 2022 | 1 | 1 |
| Deepak 2021 | 1 | 1 |
| Dihan 2023 | 1 | 1 |
| Franklin 2018 | 1 | 1 |
| Hosseini-Motlagh 2022 | 1 | 1 |
| Kaiser 2001 | 1 | 1 |
| Liu 2017 | 1 | 1 |
| Mahjoob 2023 | 1 | 1 |
| Maluleke 2024 | 0 | 1 |
| McRae 2021 | 1 | 1 |
| Meister 2023 | 0 | 1 |
| Nikzamir 2020 | 1 | 1 |
| Pellegrini 2023 | 1 | 1 |
| Sahoo 2024 | 1 | 1 |
| Thakur 2021 | 1 | 1 |
| Kappa | 0.6182 |  |
| SE | 0.2017 |  |
| Z | 3.07 |  |
| P-value | 0.0011 |  |
| Percentage agreement | 90.50% |  |

**Appendix 3: MMAT quality appraisal worksheet**

|  | | | **SCREENING QUESTIONS** | | **QUALITATIVE STUDIES** | | | | |  |
| --- | --- | --- | --- | --- | --- | --- | --- | --- | --- | --- |
| First author | Year | Title | S1. Are there clear research questions? | S2. Do the collected data allow to address the research questions? | 1.1. Is the qualitative approach appropriate to answer the research question? | 1.2. Are the qualitative data collection methods adequate to address the research question? | 1.3. Are the findings adequately derived from the data? | 1.4. Is the interpretation of results sufficiently substantiated by data? | 1.5. Is there coherence between qualitative data sources, collection, analysis and interpretation? | **Score** |
| de Campos | 2021 | A grey-DEMATEL approach for analyzing factors critical to the implementation of reverse logistics in the pharmaceutical care process | Yes | Yes | Yes | Yes | Yes | Yes | Yes | 100% |
| Deepak | 2021 | Developing an effectiveness index for biomedical waste management in Indian states using a composite indicators approach | Yes | Yes | Yes | Yes | Yes | Yes | Yes | 100% |
| Kaiser | 2001 | Solutions to health care waste: Life-cycle thinking and "green" purchasing | Yes | Yes | Yes | Yes | Yes | Yes |  | 100% |
| Liu | 2017 | Research on the reverse logistics management of medical waste based on the RFID technology | Yes | Yes | Yes | Yes | Yes | Yes | Yes | 100% |
| Pellegrini | 2023 | Sustainability of Medical Gowns Supply Chain Management in the Post-covid Era | Yes | Yes | Yes | Yes | Yes | Yes | Yes | 100% |
| Mahjoob | 2023 | Healthcare Waste and Sustainability: Implications for a Circular Economy | Yes | Yes | Yes | Yes | Yes | Yes | Yes | 100% |

|  | | | **SCREENING QUESTIONS** | | **QUANTITATIVE DESCRIPTIVE STUDIES** | | | | |  |
| --- | --- | --- | --- | --- | --- | --- | --- | --- | --- | --- |
| First author | Year | Title | S1. Are there clear research questions? | S2. Do the collected data allow to address the research questions? | 4.1. Is the sampling strategy relevant to address the research question? | 4.2. Is the sample representative of the target population? | 4.3. Are the measurements appropriate? | 4.4. Is the risk of nonresponse bias low? | 4.5. Is the statistical analysis appropriate to answer the research question? | **Score** |
| de Campos | 2021 | Logistics performance: critical factors in the implementation of end-of-life management practices in the pharmaceutical care process | Yes | Yes | Yes | Yes | Yes | Yes | Yes | 100% |
| de Campos | 2023 | End-of-use and end-of-life medicines-insights from pharmaceutical care process into waste medicines management | Yes | Yes | Yes | Yes | Yes | Yes | Yes | 100% |
| Hosseini-Motlagh | 2022 | Coordinating a socially concerned reverse supply chain for pharmaceutical waste management considering government role | Yes | Yes | Yes | Yes | Yes | Yes | Yes | 100% |
| Nikzamir | 2020 | A Healthcare Logistic Network Considering Stochastic Emission of Contamination: Bi-objective Model and Solution Algorithm | Yes | Yes | Yes | Yes | No | Yes | Yes | 80% |
| Sahoo | 2024 | Exploring Biomedical Waste Management Practices Among Healthcare Professionals: A Study From a Tertiary Care Teaching Hospital in Eastern India | Yes | Yes | Yes | Yes | No | Yes |  | 80% |
| Thakur | 2021 | Managing healthcare waste for sustainable environmental development: A hybrid decision approach | Yes | Yes | Yes | Yes | No | Yes | Yes | 80% |
| Shozib | 2025 | Management of wastes from hospitals: A case study in Pakistan | Yes | Yes | Yes | Yes | Yes | Yes | Yes | 100% |
| Ali | 2016 | Management of wastes from hospitals: A case study in Pakistan | Yes | Yes | Yes | Yes | Yes | Yes | Yes | 100% |
| Adedeji-Adenola | 2022 | Knowledge, perception and practice of pharmaceutical waste disposal among the public in Lagos State, Nigeria | Yes | Yes | Yes | Yes | Yes | Yes | Yes | 100% |
| AlAmzi | 2017 | Patients’ Knowledge and Attitude toward the Disposal of Medications | Yes | Yes | Yes | Yes | Yes | Yes | Yes | 100% |

|  | | | | | **SCREENING QUESTIONS** | | | **MIXED METHODS STUDIES** | | | | | | | | |  |
| --- | --- | --- | --- | --- | --- | --- | --- | --- | --- | --- | --- | --- | --- | --- | --- | --- | --- |
| First author | | Year | | Title | S1. Are there clear research questions? | | S2. Do the collected data allow to address the research questions? | 5.1. Is there an adequate rationale for using a mixed methods design to address the research question? | | 5.2. Are the different components of the study effectively integrated to answer the research question? | | 5.3. Are the outputs of the integration of qualitative and quantitative components adequately interpreted? | | 5.4. Are divergences and inconsistencies between quantitative and qualitative results adequately addressed? | | 5.5. Do the different components of the study adhere to the quality criteria of each tradition of the methods involved? | **Score** |
| Alshemari | 2025 | | Towards a Sustainable Solution: The Barriers and Enablers in Adopting Circular Economy Principles for Medicines Waste Management in UK and Kuwaiti Hospitals | | Yes | Yes | | Yes | Yes | | Yes | | Yes | | Yes | | 100% |
| Franklin | 2012 | | The safe insertion of peripheral intravenous catheters: a mixed methods descriptive study of the availability of the equipment needed | | Yes | Yes | | Yes | Yes | | Yes | | Yes | | Yes | | 100% |
| Meister | 2023 | | Assessing Long-Term Medical Remanufacturing Emissions with Life Cycle Analysis. | | Yes | Yes | | Yes | Yes | | Yes | | Yes | | Yes | | 100% |
| Dihan | 2023 | | Healthcare waste in Bangladesh: Current status, the impact of Covid-19 and sustainable management with life cycle and circular economy framework | | Yes | Yes | | Yes | Yes | | Yes | | Yes | | Yes | | 100% |
| McRae | 2021 | | Public Attitudes towards Medicinal Waste and Medicines Reuse in a ‘Free Prescription’ Healthcare System | | Yes | Yes | | Yes | Yes | | Yes | | Yes | | Yes | | 100% |
